# Retrospective Study of Arbovirus Circulation in Northeast Brazil in 2019 and 2022: Insights into the Re-Emergence of DENV-3 and the Co-Infection of DENV-1 and CHIKV

**DOI:** 10.3390/v17040475

**Published:** 2025-03-26

**Authors:** Sêmilly Suélen da Silva Sousa, Ana Cecília Ribeiro Cruz, Carine Fortes Aragão, Glennda Juscely Galvão Pereira Cereja, Sandro Patroca da Silva, Raira Maria Morais de Sousa, Murilo Tavares Amorim, Eliana Vieira Pinto da Silva, Bruno Tardelli Diniz Nunes, Valéria Cristina Soares Pinheiro

**Affiliations:** 1 Programa de Pós-Graduação em Biodiversidade e Biotecnologia da Rede BIONORTE, Universidade Estadual do Maranhão—UEMA, São Luis 65055-310, Brazil; semillysuelen@gmail.com; 2 Laboratório de Entomologia Médica—LABEM, Universidade Estadual do Maranhão—UEMA, Campus Caxias, Caxias 65604-380, Brazil; rayradmorais@hotmail.com; 3 Instituto Evandro Chagas, Seção de Arbovirologia e Febres Hemorrágicas, Ananindeua 67030-000, Brazil; anacecilia@iec.gov.br (A.C.R.C.); carinefaragao@gmail.com (C.F.A.); bioagl2013@gmail.com (G.J.G.P.C.); spatroca@gmail.com (S.P.d.S.); muriloamorimbio@gmail.com (M.T.A.); elianapinto@iec.gov.br (E.V.P.d.S.); brunonunes@iec.gov.com (B.T.D.N.); 4 Centro de Ciencias Biológicas e da Saúde, Universidade Estadual do Pará—UEPA, Belém 66087-662, Brazil

**Keywords:** arboviruses, coinfection, dengue, chikungunya, molecular surveillance

## Abstract

Arboviruses transmitted by *Aedes aegypti* cause high number of cases and deaths annually. The aim was to investigate the presence of the presence of Dengue (DENV), Zika (ZIKV) and Chikungunya (CHIKV) viruses in endemic areas of Maranhão, northeastern Brazil. The study was carried out in Caxias, Codó, Peritoró, and São Mateus do Maranhão in 2019 (Caxias) and 2022. The blood samples were subjected to RNA extraction and then tested by RT-qPCR. Cell culture was used to attempt viral isolation and subsequent sequencing. In total, 171 samples were analyzed (32 from 2019, 18.7%) and 72 (42.1%) were found to have arboviruses: 68 (39.7%) from Caxias; 2 (1.1%) from Codó; 1 (0.6%) from Peritoró; and 1 (0.6%) from São Mateus. Overall, 85.3% (*n* = 58) of the positive samples were infected with DENV-1, 4 (four) (5.9%) with DENV-2 (Caxias), 1 (one) (1.5%) with DENV-3 (Caxias), and in 6 (six) (7.3%) samples CHIKV was detected, with one co-infection of DENV-1 and CHIKV (Caxias). The DENV-1 genotype V and the ECSA genotype of CHIKV were characterized in samples from Caxias. The detection of DENV-1, DENV-2, DENV-3, and more CHIKV in the interior of Maranhão alerts to the importance of virological studies in these areas.

## 1. Introduction

Arthropod-borne viruses (arboviruses), which cause diseases such as dengue, yellow fever, chikungunya, and Zika, are current public health problems in tropical and subtropical areas where approximately 3.9 billion people are at risk of contracting these diseases. The periodicity and intensity of outbreaks of these diseases, especially those transmitted by *Aedes aegypti* mosquitoes, are increasing worldwide, sustained by a combination of economic, ecological, and social factors [1].

Although preventable, the situation of arboviruses, especially dengue, has worsened worldwide. Dengue is now endemic in more than 100 countries in the World Health Organization (WHO) regions of Africa, the Americas, the Eastern Mediterranean, Southeast Asia, and the Western Pacific, with the Americas, Southeast Asia, and the Western Pacific being the most severely affected regions [1]. In the Americas, according to the Pan American Health Organization (PAHO), Brazil, Argentina, Colombia, and Mexico are responsible for 90% of dengue cases and 88% of deaths, with Brazil accounting for the majority of records [2]. Although chikungunya and Zika are more under control in the country, they also present sporadic outbreaks, making arboviruses in the country a serious public health and economic burden, due to the high number of annual cases and the weakness and complications they cause in infected individuals [3].

In Brazil, arboviruses constitute a complex epidemiological scenario. The simultaneous circulation of the four serotypes of *Orthoflavivirus denguei* (dengue virus—DENV), (DENV-1, DENV-2, DENV-3 and DENV-4) may increase the risk of severe infections due to the possibility of exposure to more than one serotype, since, in an individual who has already had dengue and is subsequently infected by a different serotype, there is a greater risk of developing severe forms of the disease [4]. The four serotypes of DENV have distinct antigenic characteristics and are divided into 20 genotypes, which are distributed as follows: Genotypes I, II, III, IV, and V of DENV-1, the latter representing the majority of strains of this serotype collected in the Americas and strains from West Africa and Asia [5,6]; DENV-2 includes Asian Genotype I, Asian Genotype II, the Southeast (SE) Asian/American Genotype, the Cosmopolitan Genotype, the American Genotype, and the Wild Genotype; DENV-3 Genotypes I, II, III, IV, and V; and DENV-4 Genotypes I, II, III, and IV [7,8]. The introduction of *Alphavirus chikungunya* (chikungunya virus - CHIKV) and *Orthoflavivirus zikaense* (Zika virus - ZIKV) in 2014 and 2015, respectively, further aggravated the situation, creating an even more complex epidemiological scenario [9].

With the simultaneous circulation of different arbovirus serotypes, hyperendemicity is a critical factor due to the antibody-dependent enhancement (ADE) of the infection, a mechanism that facilitates DENV uptake by target cells, in which humoral immunity from a previous DENV infection enhances rather than inhibits viral replication, which has been identified as a key factor in severe dengue cases and related fatalities [8].

The Brazilian Ministry of Health recorded 1,545,462 cases of dengue in 2019, showing a considerable reduction in 2020 with 948,533 cases and 531,922 cases in 2021, probably due to the adoption of stricter social distancing and hygiene practices during the COVID-19 pandemic, which may have affected the spread of the mosquito and also the high underreporting of arboviruses during this period [10]. In 2022, the number of cases of arboviruses increased again, when 1,450,270 cases of dengue, 174,517 cases of chikungunya, and 9204 cases of Zika were reported [10]. Records continued to increase in 2023, when dengue reached 1,623,772 probable cases, while 54,512 and 7225 cases of chikungunya and Zika were reported, respectively [11]. In 2024, dengue notifications in the country surpassed the 6 million mark, an increase of 344.5% in the number of cases when compared to the same period of the previous year [12]. Maranhão, the state with the highest proportion of people living in poverty in Brazil, is among the states in the northeast region that has always had high records of arboviruses, especially dengue, despite efforts by the health sector to contain outbreaks of these diseases. In 2022 alone, 7369 cases of dengue, 2295 cases of chikungunya, and 296 cases of Zika were reported, with 12 deaths recorded in the state [11]. Furthermore, *Ae. aegypti*, the primary vector of these arboviruses, is widely distributed across the municipalities of Maranhão [13,14].

Another aggravating factor is the similarity of symptoms among these infections, which hinders accurate clinical diagnosis and increases the number of affected individuals [15,16]. The situation becomes even more worrying considering that there are few laboratories specialized in detecting arboviruses in the interior of Maranhão, meaning that laboratory diagnosis of suspected cases is primarily conducted in the capital, São Luís, or even outside the state, which can overburden health services and delay the results needed for disease control.

These factors highlight the need for broader use of viral detection analyses for the control and prevention of these infections in the state of Maranhão, as various studies on arboviruses have addressed the sensitivity and specificity of molecular biology techniques in human samples [15,16,17]. Therefore, this study aimed to investigate the presence of the presence of DENV, CHIKV and ZIKV in patients with suspected arbovirus infection in endemic areas of Maranhão, northeastern Brazil, using molecular biology techniques.

## 2. Materials and Methods

### 2.1. Study Area and Population

Maranhão covers an area of 331,983.29 km^2^, making it the eighth largest state in Brazil and the second largest in the northeast [18]. It is bordered to the north by the Atlantic Ocean (639.5 km), to the south and southwest by Tocantins (1060 km), to the west by Pará (798 km), and to the east and southeast by Piauí (1365 km). The state is divided into five geographical mesoregions (north, east, west, center, and south of Maranhão), which are further subdivided into 21 geographical microregions, encompassing 217 municipalities [18].

In 2022, Maranhão had an estimated population of 6,775,152 inhabitants, making it the fourth most populous state in the northeast region, and it had the lowest Human Development Index (HDI) in Brazil, at 0.676. The research was conducted in partnership with the primary, secondary, and tertiary health care networks of three municipalities in the eastern mesoregion of Maranhão: Caxias, Codó, and Peritoró. It also included São Mateus do Maranhão, located in the central region of the state. Entomological data from each municipality’s LIRAa (Rapid Index Survey for *Ae. aegypti*), conducted during the first two months of 2019, show that the municipalities are on alert for possible dengue, chikungunya, and Zika epidemics. The LIRAa index was 2.5% in Caxias, 1.0% in Codó, 1.5% in Peritoró, and 1.7% in São Mateus do Maranhão.

The municipality of Caxias is the fifth most populous in the state, with 156,973 inhabitants. Codó has an estimated population of 114,275, making it the sixth most populous municipality in Maranhão. Peritoró has 20,474 inhabitants and São Mateus do Maranhão has 38,829 [19]. In 2022, 233 cases of dengue were reported in Caxias, 47 in Codó, only 1 in Peritoró, and 18 in São Mateus [20].

### 2.2. Sampling and Ethical Procedures

The sample included 171 patients treated in the Public Health Network of the municipalities of Caxias (*n* = 149, 32 from 2019 and 117 from 2022), Codó (*n* = 4), Peritoró (*n* = 1), and São Mateus (*n* = 17) who agreed to participate in the study and met the inclusion and classification criteria for arbovirus infection established by the Ministry of Health, which relate to visits to the transmission area and the characteristic symptoms of arboviruses. They were asked to fill in the informed consent form, as well as the assent form for children and adolescents. The study was submitted to and approved by the Ethics Committee of the State University of Maranhão with the certificate of Submission for Ethical Appraisal—CSEA: 38376620.0.0000.5554.

### 2.3. Collection of Biological Material

Biological samples were collected from January to July 2019 and from January to July 2022, with a pause due to the COVID-19 pandemic. Venous blood samples were collected by percutaneous puncture, 10 mL in adults and 3 to 5 mL in children. The collection was carried out during the viremic period (up to the sixth day of the disease) by health professionals working in hospitals that routinely perform this procedure, following safety and hygiene protocols to ensure patient safety and sample quality. Only one sample of whole blood or serum was taken from each patient and stored in a hemolysis tube, labeled with the sample number. The material was obtained upon medical request for testing, which is why both whole blood and serum samples were collected. The collected material was transported to the Medical Entomology Laboratory (LABEM) at the Universidade Estadual do Maranhão (UEMA), Caxias Campus, where it was stored at −80 °C until being sent to the molecular analysis laboratory. The material was then placed in a container with liquid nitrogen and transported to the Molecular Biology Laboratory, Arbovirology and Hemorrhagic Fevers Section at the Instituto Evandro Chagas (IEC) in Pará for analysis.

### 2.4. Viral RNA Extraction and RT-qPCR

The whole blood samples were subjected to viral RNA extraction using the Trizol LS reagent (Invitrogen, Waltham, MA, USA) in association with the PureLink™ RNA Mini Kit (Invitrogen, Waltham, MA, USA), following the manufacturer’s guidelines. The QIAamp Viral RNA Mini Kit (Qiagen, Hilden, Germany) was used for the serum samples.

RT and PCR were performed in a single step using the SuperScript^®^ III Platinum^®^ One-Step qRT-PCR (ThermoFisher Scientific, Waltham, MA, USA). The DENV test involved a multiplex assay to detect the four serotypes of the virus, while the ZIKV and CHIKV tests were singleplex assays.

The assay was validated using positive controls (DENV-1–4, CHIKV, and ZIKV) and negative controls (water without nuclease). The reactions were carried out on the ABI 7500 Fast Real-Time PCR system (Applied Biosystems, Thermo Fisher Scientific, Waltham, MA, USA). The RT-qPCR assays were carried out under the following cycling conditions: an initial RT step at 50 °C for 30 min, a denaturation step at 95 °C for 2 min, 45 cycles of 15 s at 95 °C, and a final extension step at 60 °C for 1 min. Samples were considered positive when the average cycle threshold (CT) value obtained was less than 37 for DENV and less than 38 for CHIKV and ZIKV. In other words, the lower the Ct value, the higher the viral load, which may suggest a more recent or active infection.

To detect DENV, CHIKV, and ZIKV, specific primers and probes were used for dengue [19], described in Table 1, chikungunya [21] (Table 2), and Zika [22] (Table 3). Positive samples were selected for viral isolation and genomic analysis.

### 2.5. Viral Isolation in C6/36 Cells

As part of the standard test in our arbovirus investigation, thirty-seven samples from different areas, all positive by RT-qPCR, were subjected to viral isolation testing using arthropod cells (*Aedes albopictus* clone C6/36—ATCC: CRL1 660). The cells were seeded in 16 × 125 cm^2^ culture tubes containing 1.5 mL of modified Leibowitz medium with glutamine (L-15), 2.95% tryptose phosphate, non-essential amino acids, antibiotics (penicillin and streptomycin), and 2% fetal bovine serum, with the cells diluted at a 1:30 ratio. Prior to inoculation, the culture medium was discarded, and 25 µL of the sample was inoculated onto the cell monolayers. The cells were incubated at 28 °C for 60 min to facilitate the adsorption process, which was performed gently in 15 min intervals. After this period, the inoculated cells were maintained in 1.5 mL of L-15 medium, containing 2.95% tryptose phosphate, non-essential amino acids, antibiotics (penicillin and streptomycin), and 2% FBS, according to an adapted protocol [23]. The inoculated cells were kept at 28 °C and observed daily for up to 7 days or until the presence of cytopathic effect (CPE).

### 2.6. Nucleotide Sequencing

The isolates were sequenced for nucleotide analysis of the viral agent and determination of phylogenetic relationships. RNA was extracted from positive cell cultures using the QIAamp™ Viral RNA Mini Kit (Qiagen, Hilden, Germany), according to the manufacturer’s protocol.

RNA was quantified using the Qubit RNA HS Assay Kit (Thermo Fisher Scientific, Waltham, MA, USA) on Qubit 4.0 equipment, according to the manufacturer’s recommendations. The preparation of cDNA from RNA began with the synthesis of the first and second tape of cDNA, using the SuperScript™ IV VILO™ Master Mix kits (Thermo Fisher Scientific, Waltham, MA, USA) and the Second Strand cDNA Synthesis Kit (ThermoFisher), respectively. The cDNA was then purified using the Pure-Link™ PCR Purification Kit (Thermo Fisher Scientific, Waltham, MA, USA) and subsequently quantified using the Assay DNA HS Kit (Thermo Fisher Scientific, Waltham, MA, USA) on Qubit 4.0 equipment.

The genomic library was prepared according to the guidelines of the Nextera XT DNA kit (Illumina, San Diego, CA, USA) and sequenced using the NextSeq 550 platform (Illumina, San Diego, CA, USA) using the NextSeq 500/550 High Output Kit v2.5 (300 Cycles), using the paired-end methodology, according to the manufacturer’s recommendations. The genome was assembled using the De Novo Assembler methodology using the SPAdes v3.13.125 and MEGAHIT v1.2.926 programs, using k-mer values of 21, 33, 55, and 77 and 21, 31, 41, 51, 61, 71, 81, 91, and 99, respectively [24]. The contigs generated were analyzed by the DIAMOND v2.1.8 program and by BLASTX analysis using the non-redundant viral protein database (nr), considering e-value 1 × 10^−4^ and amino acid identity values.

The files referring to the contigs generated for each sample were visualized in the MEGAN v6.21.128 program using an e-value filter set to 1 × 10^−10^. The contigs that showed similarity to viral sequences of interest were visualized in the Geneious v9.1.8 program. The result of the alignment was manually inspected to make manual corrections, where necessary, using the Geneious v.9.1.8 program.

Phylogenetic inference was carried out using the nucleotide sequences of different strains of the virus available in the database of the National Center for Biotechnology Information (http://www.ncbi.nlm.nih.gov), (accessed on 28 November 2023) sing the protein-coding regions. The dataset generated together with the samples from the study was submitted to multiple sequence alignment (MSA), using the Mafft v.7 program [25]. The result of the alignment was manually inspected for corrections when necessary, using the program Geneious v.9.1.8 (https://www.geneious.com/), (accessed on 28 November 2023).

Initially, the aligned dataset was analyzed to identify the best-fitting nucleotide substitution model, which was then used to parameterize the phylogenetic reconstruction. Subsequently, phylogenetic trees were built using the maximum likelihood (ML) methodology [26]. Both methodologies were employed using the IQ-TREE v.1.6.12 program [27]. Alongside these analyses, the bootstrap test was used, establishing 1000 replicates to give greater reliability to the clade values [28]. Phylogeny visualization was performed using the FigTree v.1.4.4 program (https://github.com/rambaut/figtree/releases/tag/v1.4.4) (accessed on 28 November 2023). For the dataset used, a root sequence was not utilized; thus, the midpoint rooting methodology, available in the FigTree program, was employed. After evaluating and editing the phylogeny, a scalable vector graphics (“.sgv”) file was generated for image editing and manipulation using the Inkscape v.1.3.2 program (https://inkscape.org/release/inkscape-1.3.2/) (accessed on 28 November 2023).

## 3. Results

A total of 171 samples were analyzed by RT-qPCR, including 156 whole blood samples and 15 serum samples, from patients with suspected arbovirus infections living in the municipalities of Caxias, São Mateus, Codó, and Peritoró (Table S1). Of the total samples analyzed, 72 (42.1%) were positive, including 68 (39.7%) from Caxias—4 from 2019 and 64 from 2022. Of the four samples from the municipality of Codó, two were positive for DENV-1. One sample from Peritoró and another from São Mateus tested positive for orthoflavivirus, both from 2022 (Table 4). Four serum samples were positive.

The municipality of Caxias had the highest number of positive samples (*n* = 68; 94.4%). DENV-1 was detected in fifty-seven (79.1%) samples, CHIKV in six (8.4%), DENV-2 in four (5.5%), and DENV-3 in one (1.4%). Additionally, one co-infection with DENV-1 and CHIKV was observed in Caxias. In the other municipalities, only DENV-1 was detected (Table 1). No ZIKV RNA was detected in the samples analyzed in this study.

An attempt was made to isolate DENV-3; however, as indicated by the high CT value of 35.2 (8CX22), a low viral load was detected, making isolation and subsequent nucleotide sequencing impossible in this sample. Nevertheless, it was possible to sequence nine samples that were positive in both the RT-qPCR test and viral isolation. The complete protein-coding region/open reading frame (ORF) of the polyprotein was sequenced in four samples associated with orthoflavivirus dengue 1 (DENV-1) (GenBank accession number 132CO22: PP626439; 26CX22: PP626440; 52CX22: PP626441; and 88CX22: PP626442).

**Figure 1:**
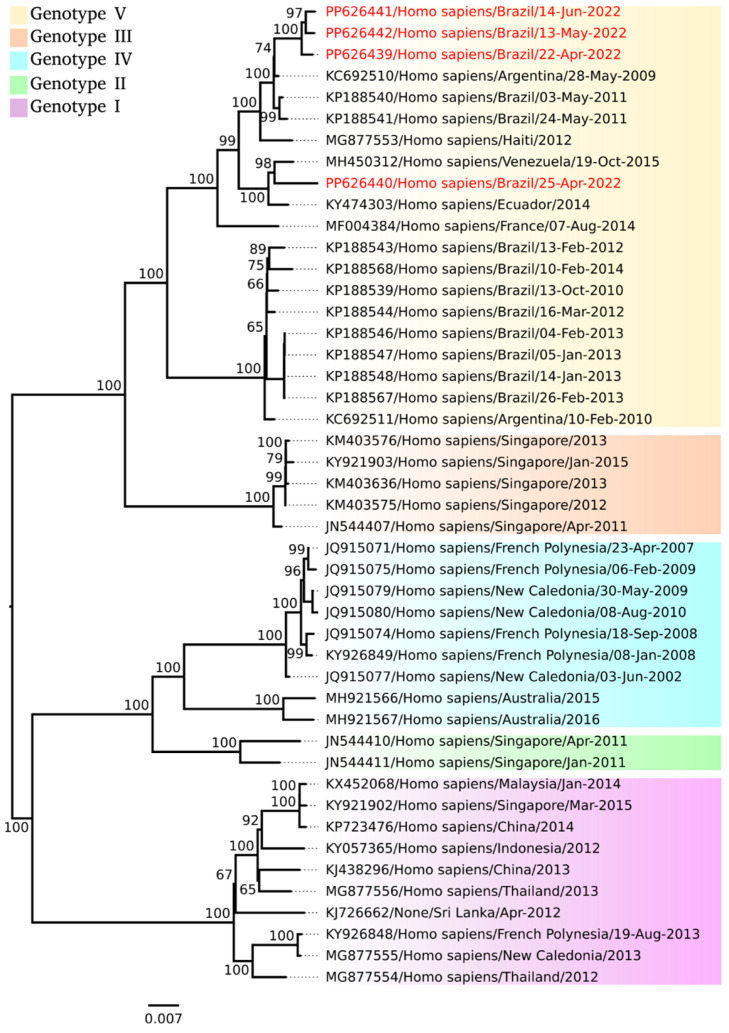
Phylogenetic tree of different virus strains belonging to DENV-1. Isolates obtained from this study are included. The maximum likelihood (ML) method was used based on the complete nucleotide sequences of the polyprotein, with the GTR+F+G4 matrix being used as the best nucleotide substitution model. Known genotypes are labeled in different colors. Samples identified in this study are highlighted in red. The numbers at each main node of the tree correspond to the bootstrap values in percentages (1000 replicates). The scale bar corresponds to the nucleotide divergence per site between sequences.

**Figure 2:**
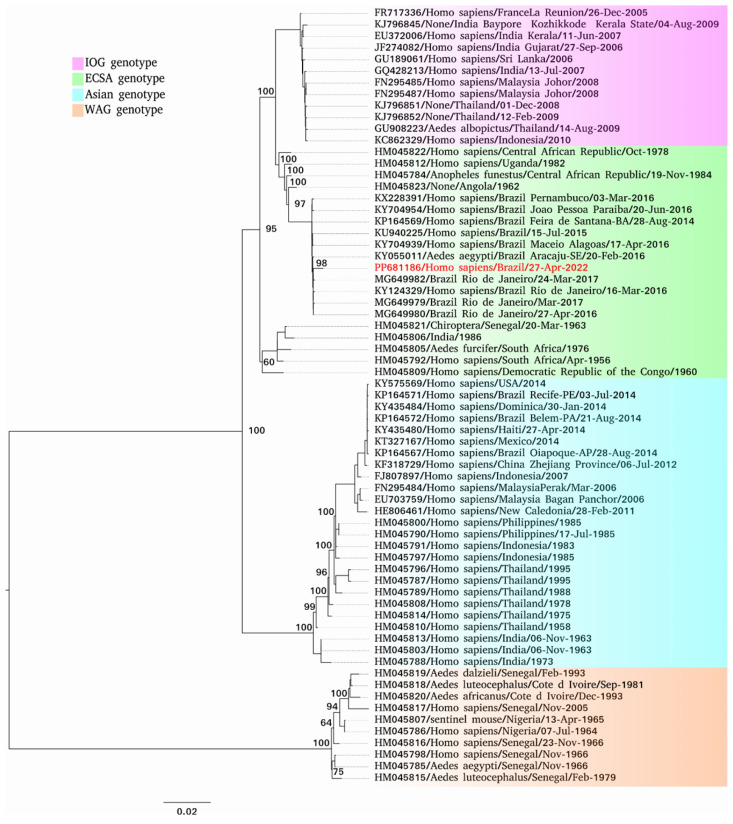
Phylogenetic tree of different virus strains belonging to CHIKV. Isolates obtained from this study are included. The maximum likelihood (ML) method was used based on the complete nucleotide sequences of the non-structural, intergenic, and structural regions, using the GTR+F+I matrix as the best nucleotide substitution model. Known genotypes are labeled in different colors. Samples identified in this study are highlighted in red. The numbers at each main node of the tree correspond to the bootstrap values in percentages (1000 replicates). The scale bar corresponds to the divergence per site between sequences.

The aligned datasets showed a positive phylogenetic signal (98.6% fully resolved trees for DENV-1 and 97.6% fully resolved trees for CHIKV) and served as the basis for building phylogenetic inferences, using the GTR+F+G4 nucleotide substitution model for DENV-1 and GTR+F+I for CHIKV. The sequences of the DENV-1 isolates were grouped into the Genotype V clade (Figure 1), while the CHIKV sequence was grouped into the Genotype ECSA clade (Figure 2).

## 4. Discussion

Arboviruses are responsible for significant epidemics of neglected tropical diseases each year in Brazil, causing major impacts, particularly on the most vulnerable populations. This study focuses on the detection of DENV-1, DENV-2, DENV-3, and CHIKV in the rural areas of Maranhão, the state with the highest concentration of people living in extreme poverty in Brazil [29]. Although ZIKV was not detected in the samples analyzed, Maranhão recorded the second highest overall positivity rate for Zika in 2024, highlighting the importance of continuous monitoring [12].

The detection of DENV and CHIKV serotypes in 2019 and 2022, periods before and after the COVID-19 pandemic, despite the gap in the intervening years, is important for predicting the risk of severe outbreaks, because when an individual who has previously had dengue is infected with another serotype, there is a higher risk of developing severe forms of the disease, such as dengue hemorrhagic fever or shock syndrome. Therefore, knowing which serotypes are circulating can assist in monitoring potential outbreaks and severe cases.

Caxias is the fifth most populous municipality in Maranhão [18]. The co-circulation of these arboviruses in the region can result in significant challenges for the health sector, leading to illness among the population and overwhelming medical care in health centers and hospitals. Additionally, illness in the population can disrupt work productivity due to the absence of affected individuals from their work activities, which contributes to the decline of the local economy. The evidence of circulation of three dengue viral serotypes in the region is concerning, as co-circulation increases the likelihood of severe disease, particularly in individuals previously infected with DENV [30]. Interestingly, the analyses also reveal a case of simultaneous DENV-1 and CHIKV infection in this municipality. Such cases are rare, but evidence suggests that this co-infection can worsen the severity of cases due to the exacerbation of symptoms [9]. Additionally, DENV-1 was identified in the municipalities of Codó, Peritoró, and São Mateus in Maranhão.

The widespread dissemination of this serotype in the state corroborates national data. Analysis of laboratory surveillance data across the country showed that the 2022 Brazilian dengue epidemic was dominated by DENV-1 in most states [10]. Genomic sequencing carried out on three samples from Caxias (Genbank accession PP626442, PP626441, and PP626440) and one sample from Codó (Genbank accession PP626439) indicated the V genotype of DENV-1. These data corroborate the findings of [31], which attested to the strong and continuous presence of this genotype in the country. Furthermore, the circulation of a new clade of DENV-1 was recently revealed in a study also conducted in the northeast of Brazil [32]. Due to the low viral load present in the DENV-2 and DENV-3 samples, it was not possible to carry out genomic sequencing on the samples positive for these serotypes in this study.

DENV-2 remained without causing epidemics in Brazil for about 10 years, but returned to the country in 2019, being identified in the state of São Paulo. In this investigation, DENV-2 was identified in the same year, but its circulation was officially confirmed in the state of Maranhão in 2022, being found in the municipalities of São Luís, São José de Ribamar, Timon, and Caxias [20].

In relation to the detection of DENV-3, it reappeared in the country in 2023, after years of not circulating in Brazil. The return of this serotype generated great concern due to the risk of a new epidemic of the disease, given the large number of people susceptible to DENV-3 infection at the time. Three autochthonous cases of DENV-3 infections (Genotype III) were detected in Roraima, and one imported case from Suriname was identified in Paraná [33].

In relation to the identification of the ECSA genotype of CHIKV (GenBank accession PP681186) in this study, it is possible to verify the maintenance of viral circulation in the state, which has been causing outbreaks throughout the country since 2014. This genotype was first reported in Brazil in 2014, in Bahia, northeast Brazil [34], and has since spread to other states such as Alagoas (2016) [35], Piauí (2016–2017) [36], and Maranhão (2017) [13], the latter recorded in the municipality of Codó. It has also been identified in the southeast, in Rio de Janeiro (2016–2018) [37], and in the north, in the state of Pará (2017) [38]. These data show that this strain is the main one circulating in the country [31].

The data from this study point to a worrying situation in the epidemiological scenario of arboviruses in the state of Maranhão in recent years. This situation requires great attention from surveillance teams to develop strategies in response to these diseases. However, the state faces a series of challenges, ranging from the wide dispersion of the *Ae. aegypti* vector, mainly due to the wide availability of deposit-type breeding sites, which are widely used by the local population for domestic activities [14,39], to the difficulties in carrying out laboratory diagnosis, which is mainly carried out in the capital, São Luís, and can lead to delays in obtaining results and, consequently, in prevention and control actions.

It is worth noting that the state of Maranhão is located in an area of great epidemiological importance in the context of arboviruses. To the west, the state borders Pará, where a wide variety of wild arboviruses circulate, such as the West Nile, Rocio, and Ilhéus viruses [40,41]. To the east, Maranhão borders Piauí, which, in addition to urban arboviruses, has been registering human cases of West Nile fever [42].

The territorial proximity of the state of Maranhão to areas endemic for wild-cycle arboviruses raises great concern due to the possibility of the introduction of other arboviruses in addition to the urban arboviruses already registered in the state, which could cause a major collapse in local health systems. The spread of these arboviruses can occur due to the constant deforestation seen in the Amazon biome, which favors the dispersal of vectors, as well as the existence of the BR-316 highway that cuts through the state of Maranhão, linking Pará (north) to the state of Alagoas (northeast) [43,44].

Recently, Maranhão has also been registering cases of another important arbovirus, Oropouche fever. As of July 2024, the Maranhão State Health Department has registered 18 cases of the disease in the state [12]. These combined factors highlight the importance of continuous monitoring and early detection of clinically significant arboviruses in Brazil and other countries to prevent epidemics and minimize the exacerbation of public health impacts [33]. This study is highly relevant to the understanding of circulating arboviruses in Maranhão, allowing us to better comprehend the complex dynamics of virus co-circulation in the state, as well as the correlation of the identified arboviruses with those circulating in other regions of the country. The findings may play a key role in guiding timely management and control measures during future viral outbreaks to prevent potential impacts on public health and the overloading of health units.

## 5. Conclusions

The research allowed us to understand the circulation of important arboviruses in the interior of Maranhão, in northeastern Brazil. The data reflect the situation of arboviruses immediately before and after the COVID-19 pandemic, which may be crucial for identifying possible variations in the dynamics of these diseases due to this period. The detection of three dengue serotypes, the V genotype of DENV-1, and the ECSA genotype of CHIKV, as well as the co-infection between DENV and CHIKV, which has yet to be fully elucidated, highlights the relevance of viral monitoring studies in endemic regions. The detection of DENV-3 emphasizes the need for continuous epidemiological surveillance to determine whether this finding truly reflects a pattern of re-emergence of this serotype in the area. Knowledge of the actual situation of DENV and CHIKV in eastern Maranhão calls for further studies, particularly regarding possible transmission routes to the north and other areas of northeastern Brazil. Virological research plays a crucial role in characterizing potential scenarios and assessing the impact on public health during future outbreaks, especially considering the replacement of DENV serotypes and the co-circulation of different arboviruses.

## Figures and Tables

**Table 1:** Primers and probes for the RT-qPCR assay for DENV detection.

Viral Serotype	Nucleotide Sequence (5′–3′)	Frequency	Genomic Position
DENV-1 F	CAAAAGGAAGTCGYGCAATA	48/48	8936–8955
DENV-1 R	CTGAGTGAATTCTCTCTGCTRAAC	48/48	9023–9047
Probe DENV-1	FAM-CATGTGGYTGGGAGCRCGC-BHQ1	48/48	8961–8979
DENV-2 F	CAGGCTATGGCACYGTCACGAT	40/50	1426–1447
DENV-2 R	CCATYTGCAGCARCACCATCTC	48/50	1482–1504
Probe DENV-2	VIC—CTCYCCRAGAACGGGCCTCGACTTCAA-BHQ1	43/50	1454–1480
DENV-3 F	GGACTRGACACACGCACCCA	50/51	701–720
DENV-3 R	CATGTCTCTACCTTCTCGACTTGYCT	51/51	749–775
Probe DENV-3	Texas Red–ACCTGGATGTCGGCTGAAGGAGCTTG-BHQ2	50/51	722–747
DENV-4 F	TTGTCCTAATGATGCTRGTCG	17/18	884–904
DENV-4 R	TCCACCYGAGACTCCTTCCA	18/18	953–973
Probe DENV-4	Cy5–TYCCTACYCCTACGCATCGCATTCCG-BHQ2	18/18	939–965

Source: [19].

**Table 2:** Primers and probes for the RT-qPCR assay for CHIKV detection.

**Name**	**Nucleotide Sequence (5′–3′)**	**Genomic Position**
CHIKV NSP1 874F	AAAGGGCAAACTCAGCTTCAC	874–894
CHIKV NSP1 961R	GCCTGGGCTCATCGTTATTC	961–942
CHIKV NSP1 899p	FAM-CGCTGTGATACAGTGGTTTCGTGTG	899–923

Source: [21].

**Table 3:** Primers and probes for the RT-qPCR assay for ZIKV detection.

**Name**	**Nucleotide Sequence (5′–3′)**	**Genomic Position**
ZIKV NS5 F	AARTACACATACCARAACAAAGTG G	9271–9297
ZIKV NS5 R	TCCRCTCCCYCTYTGGTCTTG	9352–9373
Probe ZIKV	FAM—CTYAGACCAGCTGAAR-BBQ	9304–9320

Source: [22].

**Table 4:** Number and percentage value of positive clinical samples per municipality of origin and by detected arbovirus.

Municipality	DENV-1 (N/%)	DENV-2 (N/%)	DENV-3 (N/%)	CHIKV (N/%)	Samples Positive
Caxias	57 (79.1%)	4 (5.5%)	1 (1.4%)	6 (8.4%)	68 (94.4%)
Codó	2 (2.8%)	0	0	0	2 (2.8%)
Peritoró	1 (1.4%)	0	0	0	1 (1.4%)
S. Mateus	1 (1.4%)	0	0	0	1 (1.4%)
Total	61 (84.7%)	4 (5.5%)	1 (1.4%)	6 (8.4%)	72 (100%)

## Data Availability

The data is contained in the article and Supplementary Materials.

## Supplementary Materials

The following supporting information can be downloaded at: https://www.mdpi.com/article/10.3390/v17040475/s1, Table S1: Samples analyzed and identification of CT values obtained in positive samples from the municipalities of Caxias, Codó, Peritoró and São Mateus, Maranhão, Brazil.
